# Highly Transparent, Self-Healing, and Self-Adhesive Double Network Hydrogel for Wearable Sensors

**DOI:** 10.3389/fbioe.2022.846401

**Published:** 2022-02-07

**Authors:** Kai Chen, Mingxiang Liu, Feng Wang, Yunping Hu, Pei Liu, Cong Li, Qianqian Du, Yongsheng Yu, Xiufeng Xiao, Qian Feng

**Affiliations:** ^1^ Fujian Provincial Key Laboratory of Advanced Materials Oriented Chemical Engineering, College of Chemistry and Materials Science, Fujian Normal University, Fuzhou, China; ^2^ School of Resources and Chemical Engineering, Sanming University, Sanming, China; ^3^ Department of Biomaterial, College of Life Sciences, Mudanjiang Medical University, Mudanjiang, China; ^4^ Chongqing Institute of Green and Intelligent Technology, Chinese Academy of Sciences, Chongqing, China; ^5^ Key Laboratory of Biorheological Science and Technology, Ministry of Education College of Bioengineering, Chongqing University, Chongqing, China

**Keywords:** double network hydrogel, transparent, self-healing, adhesive, wearable strain sensor

## Abstract

Hydrogel-based flexible electronic devices are essential in future healthcare and biomedical applications, such as human motion monitoring, advanced diagnostics, physiotherapy, etc. As a satisfactory flexible electronic material, the hydrogel should be conductive, ductile, self-healing, and adhesive. Herein, we demonstrated a unique design of mechanically resilient and conductive hydrogel with double network structure. The Ca^2+^ crosslinked alginate as the first dense network and the ionic pair crosslinked polyzwitterion as the second loose network. With the synthetic effect of these two networks, this hydrogel showed excellent mechanical properties, such as superior stretchability (1,375%) and high toughness (0.57 MJ/m^3^). At the same time, the abundant ionic groups of the polyzwitterion network endowed our hydrogel with excellent conductivity (0.25 S/m). Moreover, due to the dynamic property of these two networks, our hydrogel also performed good self-healing performance. Besides, our experimental results indicated that this hydrogel also had high optical transmittance (92.2%) and adhesive characteristics. Based on these outstanding properties, we further explored the utilization of this hydrogel as a flexible wearable strain sensor. The data strongly proved its enduring accuracy and sensitivity to detect human motions, including large joint flexion (such as finger, elbow, and knee), foot planter pressure measurement, and local muscle movement (such as eyebrow and mouth). Therefore, we believed that this hydrogel had great potential applications in wearable health monitoring, intelligent robot, human-machine interface, and other related fields.

## Introduction

Conventional semiconductor-based strain sensors have limitations in the next generation of electronics due to their inherent deficiencies (Liu et al., 2020), including brittleness, rigidity, and low biocompatibility (Zhang et al., 2022). In recent years, the applications of conductive hydrogel in wearable devices, soft robots, and artificial skin have attracted researchers’ attention (Shen et al., 2021; Sun et al., 2021; Zhu et al., 2021). Among these application scenarios, conductive hydrogel as wearable strain sensor for human movement monitoring is one of the most studied and reported directions (Ma et al., 2021; Yu et al., 2021; Zhang et al., 2021; Yang et al., 2022a). According to previous reports, the wearable strain sensor should be able to attach to human skin comfortably and monitor human motions with wide sensing range, high precision and good durability (Fan et al., 2018; Nam et al., 2020). In addition, it also must meet the requirements of flexibility, low power consumption, biocompatibility, and portability. In order to meet all of these demands, a series of multifunctional conductive hydrogels have been prepared through the precise design of the hydrogel component and internal network structure (Chimene et al., 2020).

Hydrogel is a kind of viscoelastic material with three-dimensional network structure, which can absorb and retain a large amount of water (Dai et al., 2020; Shen et al., 2021). It is usually composed of chemically or physically crosslinked hydrophilic polymers (Li W. et al., 2021). Ionic conductive hydrogels are considered as promising candidates for the development of flexible strain sensors with large strain ranges and high sensitivity (Liu et al., 2018; Zhou et al., 2019). It has a unique porous structure and can provide an effective channel for ion transport (Zhao X. et al., 2019), so it has high ionic conductivity. At the same time, they have high flexibility, transparency, and good biocompatibility (Luo et al., 2021; Ding et al., 2022). Although highly tensile and self-healing ionic hydrogels are usually reported, most synthetic ionic hydrogels exhibit strain-softening properties (Peng et al., 2022; Zhou et al., 2022). For stretchable ionic hydrogels, there are often conflicts between tensile properties, self-healing properties and high mechanical strength (Yang et al., 2022b; Zhao et al., 2022). In general, good tensile properties, self-healing property, and self-adhesion are favorable for the use of hydrogels in wearable devices (Chen Z. et al., 2021). Excellent tensile property makes hydrogel-based wearable devices suitable for large strain of human body, thus expanding the application range of sensors (Sun et al., 2021). He et al. designed a silk fibroin-based hydrogel with considerable tensile and compressive properties, which enabled it to be assembled as a strain/pressure sensor with a wide sensing range (>600%) (He et al., 2020). The self-healing property can increase the service life of the device, which is critical to improve the durability of flexible wearable sensing devices (Pei et al., 2021). Lin et al. synthesized a multifunctional biomimetic hydrogel inspired by natural skin for use in wearable devices, achieving high self-healing (98.6% in 10 min) (Lin et al., 2019). The self-adhesive hydrogel can establish a stable and reliable interface with the measured target and improve the detection of weak and physiological signals (Jin et al., 2021). Wu et al. constructed a mussel-inspired PDA/BT/PAA glycerol-hydrogel (G-hydrogel), which exhibited wonderful self-adhesive performances (adhesion strength to porcine skin of 18 kPa) (Wu et al., 2020). Therefore, we tend to find solution from the double network hydrogel system with outstanding mechanical properties.

Double network hydrogel is proposed by Gong Jianping group (Wen et al., 2020). Unlike traditional single-network hydrogels, double network hydrogels are composed of two different polymer networks with asymmetric structure (Wang T. et al., 2022; Kim et al., 2022), including a rigid and brittle first network and a soft and stretchable second network (Ahmed et al., 2020; Kim et al., 2020). The toughening mechanism of the double network structure is mainly based on the “sacrificial bond theory” (Sun et al., 2013). When an external force is applied to the hydrogel, the first network is disconnected to effectively dissipate the energy and protects the second network, which can maintain pressure and store elasticity to strengthen the hydrogel (Ling et al., 2021; Xu et al., 2022; Yao et al., 2022). Studies based on this theory have succeeded in producing tough hydrogels that are partially or completely self-healing after internal ruptures (Kim et al., 2022). These hydrogels combine the advantages of conductive medium and three-dimensional hydrogels, such as softness, self-healing property, adhesivity, biocompatibility, and electrical-responsive, which are beneficial to their applications in flexible wearable strain sensors (El-Atab et al., 2020; Feng et al., 2022). Therefore, the design of the double network hydrogel provides a new thinking for the development of wearable electronic materials (Qin et al., 2020; Li X. et al., 2021).

**FIGURE 1:**
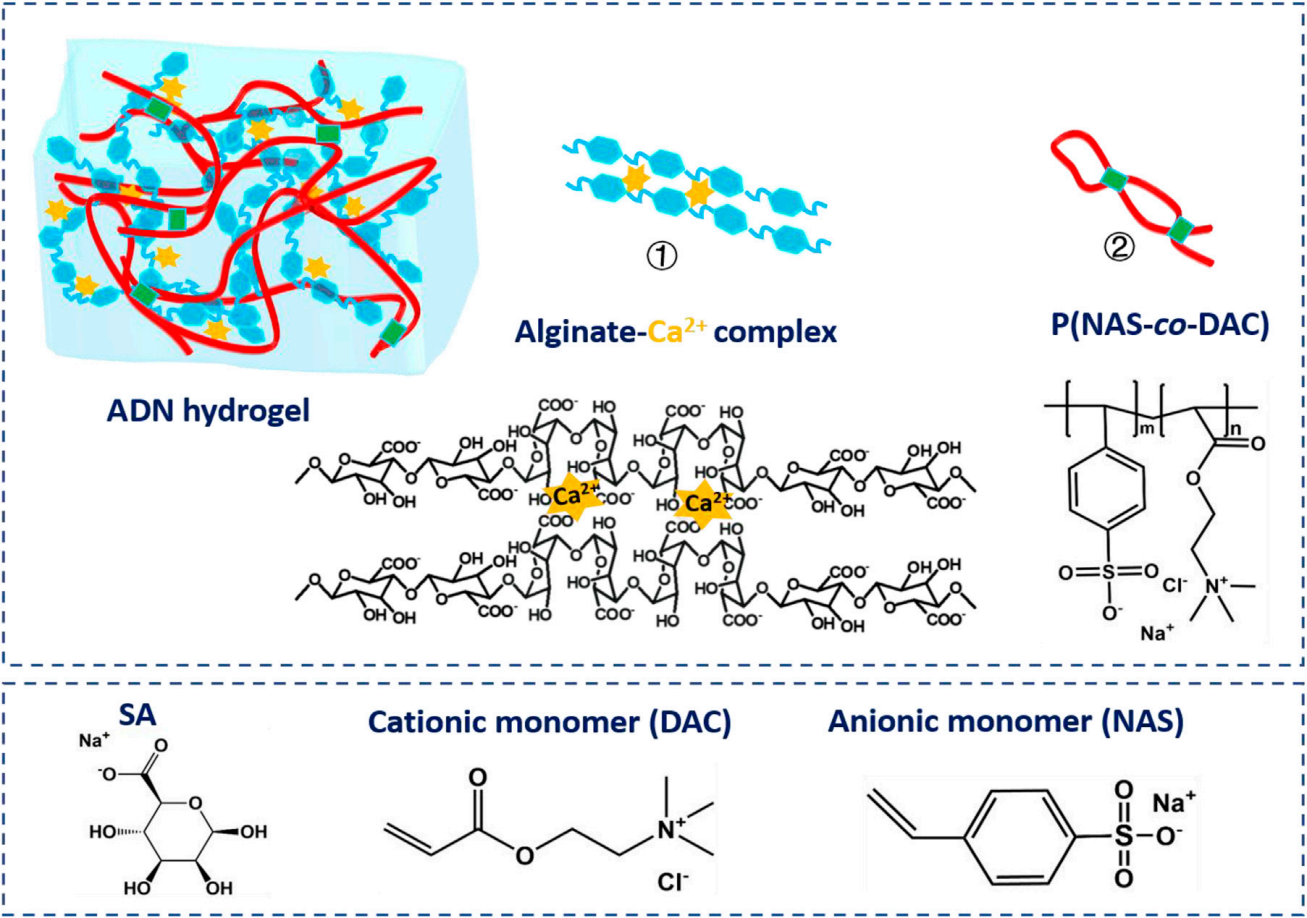
Schematic of the preparation process of the ADN hydrogel.

In this work, a novel conductive double network hydrogel was prepared by a one-pot and two-step procedure. This hydrogel was synthesized by an alginate network physically cross-linked by calcium ions and interpenetrating copolymers consisting of anionic monomer sodium p-styrene sulfonate (NAS) and cationic monomer acryloxyethyl trimethyl ammonium chloride (DAC) (Figure 1). The reversible physical cross-link brought advantages such as energy dissipation, super elasticity, and adaptive self-adhesion due to the ion dipole or dipole-dipole interaction created by strong dipolar zwitterionic units (Huang et al., 2021; Mirzaei et al., 2021). The obtained hydrogels were denoted as ADN, where A refers to sodium alginate, D refers to DAC, N refers to NAS. This hydrogel showed remarkable stretchability (∼1,375%), toughness (0.57 MJ/m^3^), high optical transmittance (∼92.2%, Supplementary Figure S1), and self-adhesion to diverse substrates. The hydrogels also present high ionic conductivity (0.25 S/m) and sensitivity (up to 3.21 of the gauge factor for the tensile strain responses). Our research also demonstrated that our ADN hydrogel could work well as a wearable strain sensor directly to respond to a variety of large joint flexion (such as finger, elbow and knee) and local muscle movement (such as eyebrow and mouth). All of these verified its great potential in personalized healthcare monitoring, human–machine interfaces, and artificial intelligence.

## Experimental Section

### Materials

Sodium p-styrene sulfonate (NAS), acryloxyethyl trimethyl ammonium chloride (DAC), 2-azobis (2-methyl-propionamidine) dihydrochloride (V-50, 98%), D-(+)-gluconic acid δ-lactone (GDL), ethylenediaminetetraacetic acid calcium disodium salt hydrate (EDTANa_2_Ca), sodium alginate, and they were purchased from Sinopharm Chemical Reagent Co., LTD. All the chemicals were purchased commercially and used directly without further purification. Deionized water (DI water) was used in all the experiments.

### Preparation of ADN Hydrogel

The ADN hydrogel was prepared by a one-pot/two-step method. In the synthesis process, sodium alginate (3 wt%) aqueous solution was prepared at 60°C. After the addition of EDTANa_2_Ca, NAS, DAC and initiator V-50 were added into the solution immediately. The mixture was stirred for another 30 min to form a thick solution. The formulation is shown in Supplementary Table S1. All the solutions were centrifugally degassed at 5,000 r/min. Then GDL was added into the solution to trigger the release of the Ca^2+^ to crosslink the alginate. After centrifugally degassing at 3,000 r/min, the mixed precursor solution was transferred to a mold. The precursor was stored at room temperature for 3 h to form the alginate network crosslinked by Ca^2+^ ions. Finally, the precursor was placed in a water bath of 45°C for 12 h to initiate the *in-situ* copolymerization of NAS and DAC. Hydrogels with different NAS contents and sole calcium alginate hydrogels (Alg) were prepared (Supplementary Table S1).

### Characterization

The crystalline nature of the hydrogels was analyzed by X-ray diffraction (Japanese Ultima IV) with copper as the target and operating voltage range of 20–60 kV. The sample was placed horizontally with a scanning range of 5°–80° and a scanning speed of 20°/min. FEI Inspect F50 scanning electron microscopy (SEM) was used to analyze the microstructure of the composite hydrogel. The hydrogel was treated with liquid nitrogen to expose its internal structure. The freeze-dried hydrogel sample was mounted on a copper stud and coated with gold/palladium sputtering for 60 s. The infrared absorption spectra of the prepared hydrogels were measured using a Fourier Transform infrared (FTIR) spectrometer (Nicolet 5,700) in transmission mode under potassium bromide pellets. All spectra were obtained by 16 scans in the range of 4,000–500 cm^−1^. The transmission spectrum of hydrogel with thickness of 1.6 mm in the wavelength range of 800–400 nm was characterized by UV-visible spectrophotometer.

### Mechanical Testing of the Hydrogels

The mechanical properties of the hydrogels were investigated using an electrical universal material testing machine (WDW-05) at room temperature. For tensile tests, the samples were prepared as the cylindrical-shaped strip with a 40 mm of length and 2.8 mm of diameter and stretched with a strain rate of 60 mm/min. For compression tests, a cylindrical hydrogel sample with a diameter of 10 mm and a height of 15 mm was placed on the bottom plate and the top plate was compressed at a strain rate of 10 mm/min. The adhesion strength of the hydrogels on different kinds of surfaces were performed by using a universal material testing machine. Each sample was tested at least three times and the results were reported with an average standard deviation. The adhesion strength was calculated by dividing the maximum force by the overlapping area of the adhesive position.

### Electrical Measurements of the Hydrogels

The resistivity of the hydrogels was measured by a digital four-probe tester (Suzhou Crystal Lattice). Resistance change of the hydrogels with mechanical deformation was tested by LCR meter (TH 2832). The two ends of hydrogels were inserted into copper wires to connect the LCR meter, and the changes of hydrogels resistance were recorded in real time.

### Fabrication and Testing of the Hydrogel-Based Flexible Wearable Strain Sensor

During the preparation of the hydrogel-based wearable strain sensor, the ADN hydrogel was made into strips of 30 mm × 8 mm × 1.5 mm in size. Two conductive copper sheets with conducting wires were tightly fixed on the two ends of the sample to assemble the strain sensor. The ADN hydrogel was sandwiched between two medical PU tapes. The function of PU tapes was mainly to prevent the evaporation of water in the hydrogel. During the monitoring of human movements, the wearable strain sensor was directly attached to the volunteers’ skin, and the real-time change of the resistance of the sensor was recorded with an LCR meter. All experiments for monitoring human movements were performed with the consent of the volunteers. The experimental scheme was approved by the Human Experimental Ethics Committee of Fujian Normal University (Approve No.20200039).

### Statistical Analysis

All the data were expressed as the means standard deviations (SD). Statistical analysis was executed with Student’s t-test. If the *p*-value was lower than 0.05, the difference was considered significant.

## Results and Discussion

### Design Principles and Material Synthesis

**FIGURE 2:**
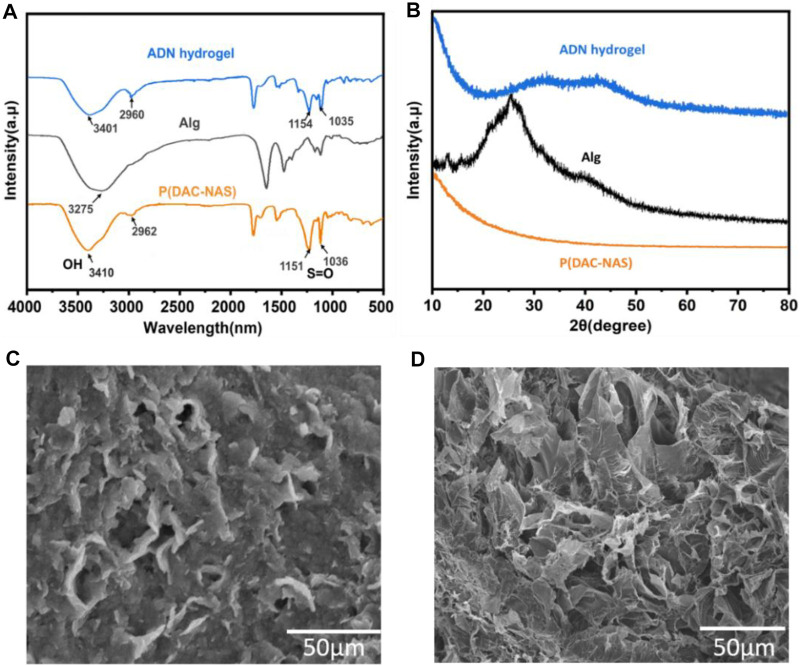
Characterization of the hydrogels. **(A)** FTIR spectra of Alg, P (DAC-co-NAS) and ADN_1.5_ hydrogels. **(B)** XRD pattern of Alg, P (DAC-co-NAS) and ADN_1.5_ hydrogels. SEM images of Alg **(C)** and ADN_1.5_ hydrogels **(D)**.

In the ADN double network hydrogel system, alginate and Ca^2+^ were physically cross-linked to form the first layer network, and anionic monomer NAS and cationic monomer DAC were polymerized to form the second layer network via ionic pair. Naturally derived alginate contains consecutive or alternating (1,4)-linked β-d-mannuronate (M) blocks and α-l-guluronate (G) blocks, where the adjacent G blocks can chelate divalent or multivalent metal ions to form an ionic crosslinking network. In order to avoid the formation of a heterogeneous network due to the rapid ion release process, we chose the (EDTANa_2_Ca)/D-(+)-gluconic acid δ-lactone (GDL) system to delay the Ca^2+^ release during the alginate-Ca^2+^ crosslinking formation (Akay et al., 2017; Yang et al., 2021). Sulfonic anions of NAS interact with ammonium ions via electrostatic forces to produce the second physical crosslinking network. Furthermore, the rich hydroxyl and carboxyl groups in alginate trigged slightly crosslinking between the two networks with hydrogen bonds and electrostatic interactions, which could be determined by the shift of the O-H and S=O peaks in the FTIR (Figure 2A) (Ilcikova et al., 2015). The XRD analysis on the samples was performed to further evaluate the impact of physical blending conditions on crystalline structure as shown in Figure 2B. The diffractogram of Alg presented a broad peak at 2θ = 24, indicating its amorphous nature (Feng et al., 2022). An explicit decrease in the crystallization of alginate was observed in the XRD spectrum of the ADN_1.5_ hydrogel, which might be contributed to its high optical transparency. SEM images of the morphology of Alg hydrogel, ADN_1.5_ hydrogel, and P(NAS-co-DAC) hydrogel were presented in Figure 2C,D and Supplementary Figure S2, respectively. As could be seen from Figure 1A, the surface of the Alg hydrogel was rough and dense without obvious porous structure. This might be due to the dense structure formed by sufficient cross-linking between alginate and calcium ions in Alg hydrogels (Qu et al., 2016). This structure also led to the hard and brittle properties of Alg hydrogels (Zhao Y. et al., 2019). As could be seen from Supplementary Figure S2, the P(NAS-co-DAC) hydrogel had obvious porous structure, the hole was large and the structure was loose. Whereas, the surface of the ADN_1.5_ hydrogel exhibited a reticulate structure (Figure 1C), which was highly beneficial to its toughness and stretchability (Zeng et al., 2020). This difference might be caused by the insufficiently cross-linking of the calcium alginate network in ADN_1.5_ hydrogels due to the introduction of P(NAS-co-DAC) network, which showed a looser structure than Alg hydrogels and a denser structure than P(NAS-co-DAC) hydrogel.

### Mechanical Properties of the Hydrogels

**FIGURE 3:**
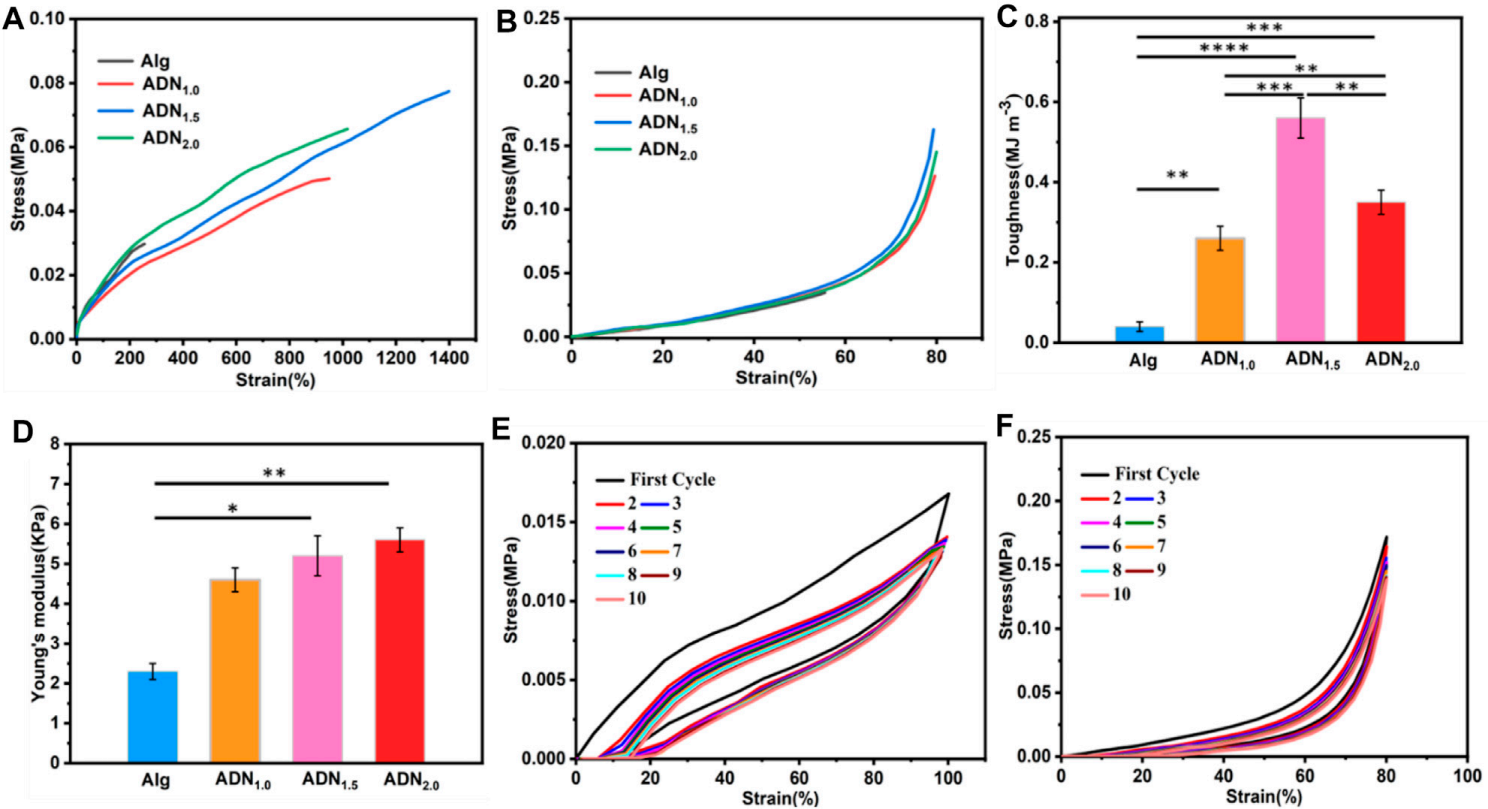
Mechanical properties of the ADN hydrogels. **(A)** Tensile strain vs. stress curves of the ADN hydrogels. **(B)** Compressive strain vs. stress curves of the hydrogels at 80% strain. Toughness **(C)** and Young’s modulus **(D)** of the hydrogels. **(E)** Ten successive cyclic tensile loading−unloading curves of the ADN_1.5_ hydrogel at 100% strain. **(F)** Ten successive cyclic compressive tests of the ADN_1.5_ hydrogel at 80% strain. ****p < 0.0001, ***p < 0.001, **p < 0.01, *p < 0.05.

The mechanical properties of Alg hydrogel were significantly enhanced by introducing the second P (DAC-co-NAS) network. As shown in Figure 3A, the tensile properties of ADN hydrogel were greatly improved compared with Alg hydrogel. In terms of the hydrogel stretchability, the ADN_1.5_ hydrogel exhibited the maximum tensile fracture length of 1,375%, which was superior to reported hydrogels in some literatures. On the contrary, the Alg hydrogel presented a fracture tensile strain of only 212%. A similar result also presented in the following compressive test (Figure 3B). Furthermore, compared with Alg hydrogel, the elastic modulus and toughness of ADN hydrogel were also significantly increased (Figure 3C,D). Photographs of the mechanical performances of the ADN hydrogels were shown in Supplementary Figure S3. Herein, in view of the excellent stretchability and toughness of the ADN_1.5_ hydrogel, we selected it as the best group for further test. Figure 3E showed 10 tensile-relaxation cycles at 100% strain. The tensile strength decreased after the first tensile cycle due to the inevitable viscosity of the polymer matrix and some permanently broken chemical bonds (Liu et al., 2021; Xue et al., 2021). The subsequent coincident tensile cycles, indicating that the reversible bond fracture and recombination had high repeatability and stability. As for the compressive cycle experiment, 10 cycles were basically consistent with the stress curves, which strongly proved that the hydrogel had good elasticity and fatigue resistance (Figure 3F). We speculated that it was because that compression was difficult to cause irreversible dissociation of the hydrogel crosslinking.

**FIGURE 4:**
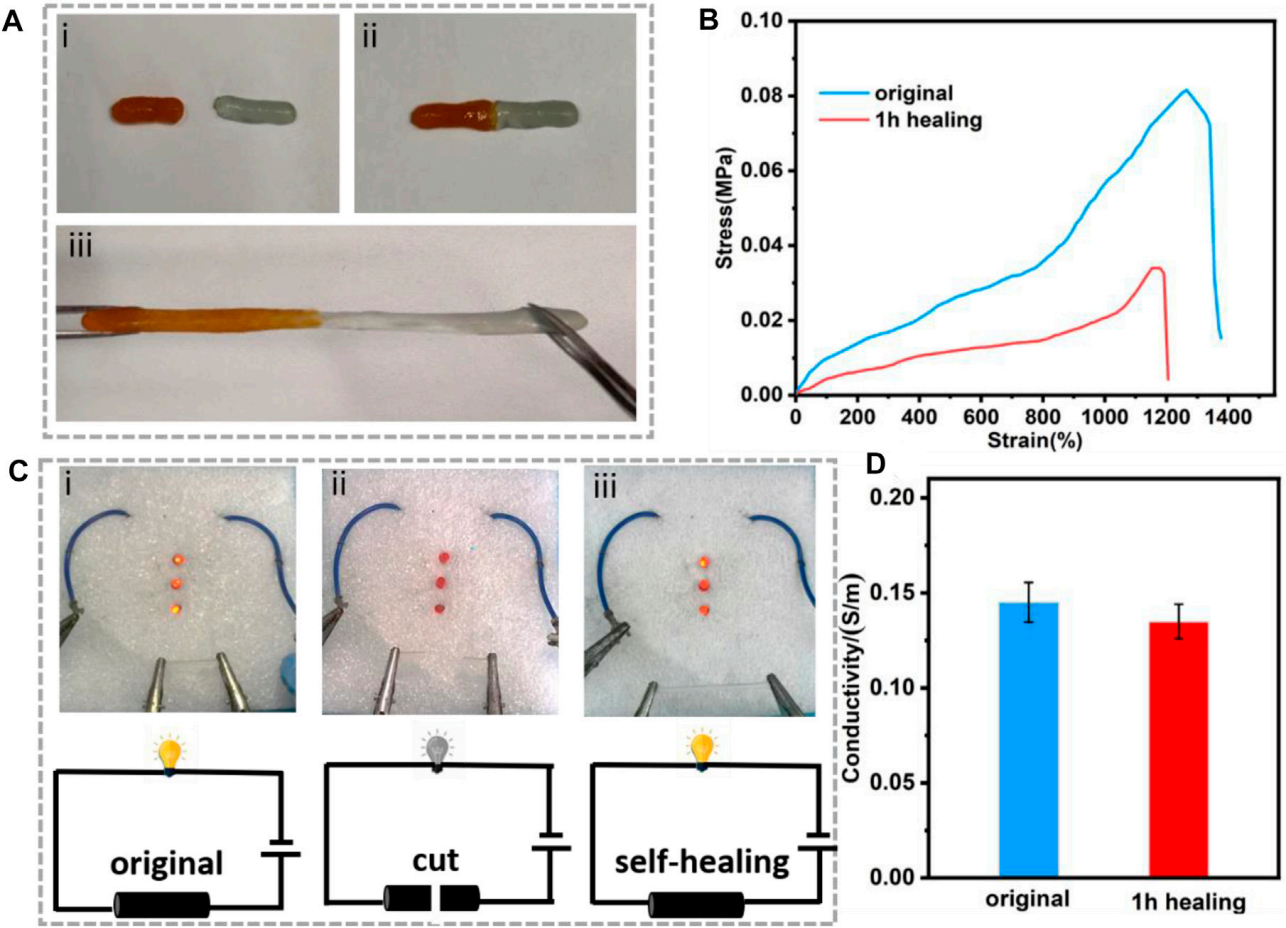
**(A)** The self-healing behavior of the ADN_1.5_ hydrogel: (i) original hydrogel, (ii) completely broken hydrogel, (iii) stretched hydrogel after self-healing. **(B)** Tensile strain vs. stress curves of the hydrogel after self-healing. **(C)** A circuit consisting of the hydrogel in series with red LED bulbs: (i) original hydrogel, (ii) completely broken hydrogel, (ii) self-healed hydrogel, and the corresponding schematic diagrams of the circuit. **(D)** The conductivity of the ADN_1.5_ hydrogel after 1 h healing.

The ADN hydrogel exhibited the ability of self-healing due to its dynamic crosslinking (Chen G. et al., 2021; Huang et al., 2021). As shown in Figure 4A **,** the ADN_1.5_ hydrogel was cut in half with a razor blade and then reassembled. After 30 min at room temperature, we observed that the two parts had healed into complete one, which still showed good stretchability (Figure 4B). The self-healing rate is defined as ɛ = E/E_0_, where E_0_ is the initial elongation at break and E is the elongation at break after self-healing (Deng et al., 2022). After calculation, the self-healing rate of composite hydrogel reached 86.5%. As shown in Figure 4C, the ADN_1.5_ hydrogel was connected to LED beads by a 6V power supply. After cutting the ADN_1.5_ hydrogel in half with a razor blade, the LED beads went out. After combining the two bifurcated parts together and repairing the dynamic cross-linking between the contact surfaces, the LED beads lit up again. The conductivity of the hydrogel after 1 h self-healing was 93% of the original hydrogel (Figure 4D). Due to the excellent self-repair ability of the hydrogel, the service life of the hydrogel as a flexible electronic device should be prolonged, leading to the great application advantages in flexible wearable strain sensors.

### Adhesive Properties of the Hydrogels

**FIGURE 5:**
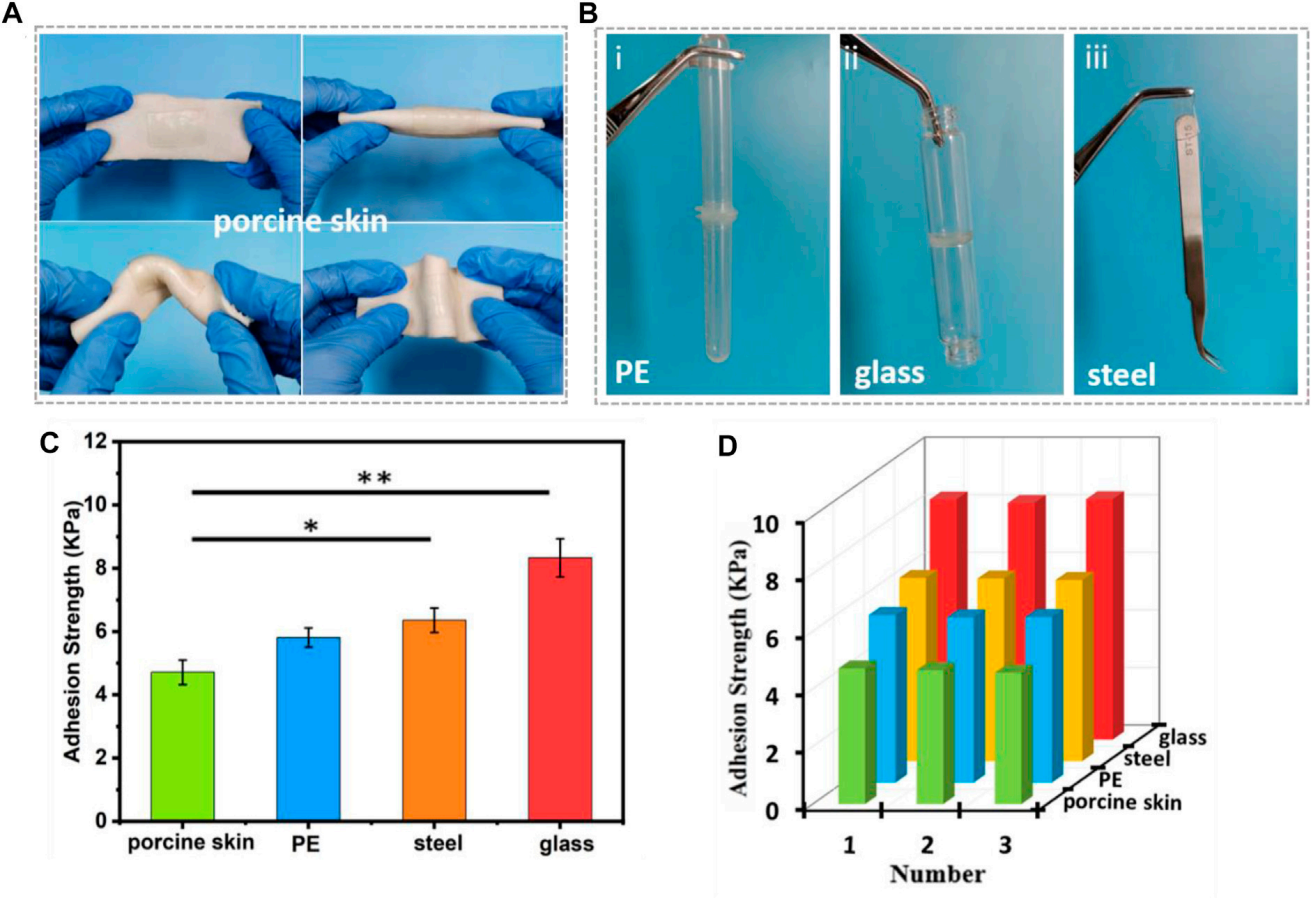
Adhesion performance of the ADN_1.5_ hydrogels. Photos of the self-adhesive hydrogels attached on porcine skin. **(A)** Photos of the self-adhesive hydrogels attached on porcine skin. **(B)** Photos of the self-adhesive hydrogels attached on PE (i), glass (ii), stainless steel (iii). **(C)** Adhesive strength of the ADN_1.5_ hydrogels to different substrates. **(D)** Repeatable self-adhesive behavior of the hydrogels to different substrates. **p < 0.01, *p < 0.05.

Mechanical compliance and durability are key factors for signal transmission of strain sensor, and conformation adhesion can adaptively overcome interfacial gaps and improve the sensitivity of signal acquisition (Choi et al., 2018). The ADN_1.5_ hydrogel could adhere to different kinds of surfaces (such as porcine skin, PE, glass, etc.). The conformal adhesion of the ADN_1.5_ hydrogel to porcine skin was shown in Figure 5A. The ADN_1.5_ hydrogel adhered tightly to porcine skin under folding and twisting, making it difficult to peel off. The adhesion photographs and adhesion strength of the hydrogels on other substrates were shown in Figures 5Bi-iii,C, respectively. This adhesion was attributed to the high polarity of the zwitterionic polymer in the hydrogel (Asha et al., 2021; Yu and Wu, 2021). Both charged groups (cationic quaternary ammonium and anionic sulfonate) and polar groups (S=O) tend to interact with other charged or polar groups on the surface of most substrates via ion dipole and/or dipole-dipole interactions, resulting in a strong interfacial bonding (Kim et al., 2021). This inherent adhesive property enabled the ADN hydrogels to be applied in human-computer interaction, soft robotics and other fields in a fitting manner. In addition, the cyclic adhesion test of ADN_1.5_ hydrogel on different substrates showed that the adhesion of the hydrogel remained stable after multiple use (Figure 5D). This multi-cycle adhesion made the ADN hydrogel an ideal material for building wearable strain sensors of easy use and economical.

### Electronic Performance of the Hydrogel

**FIGURE 6:**
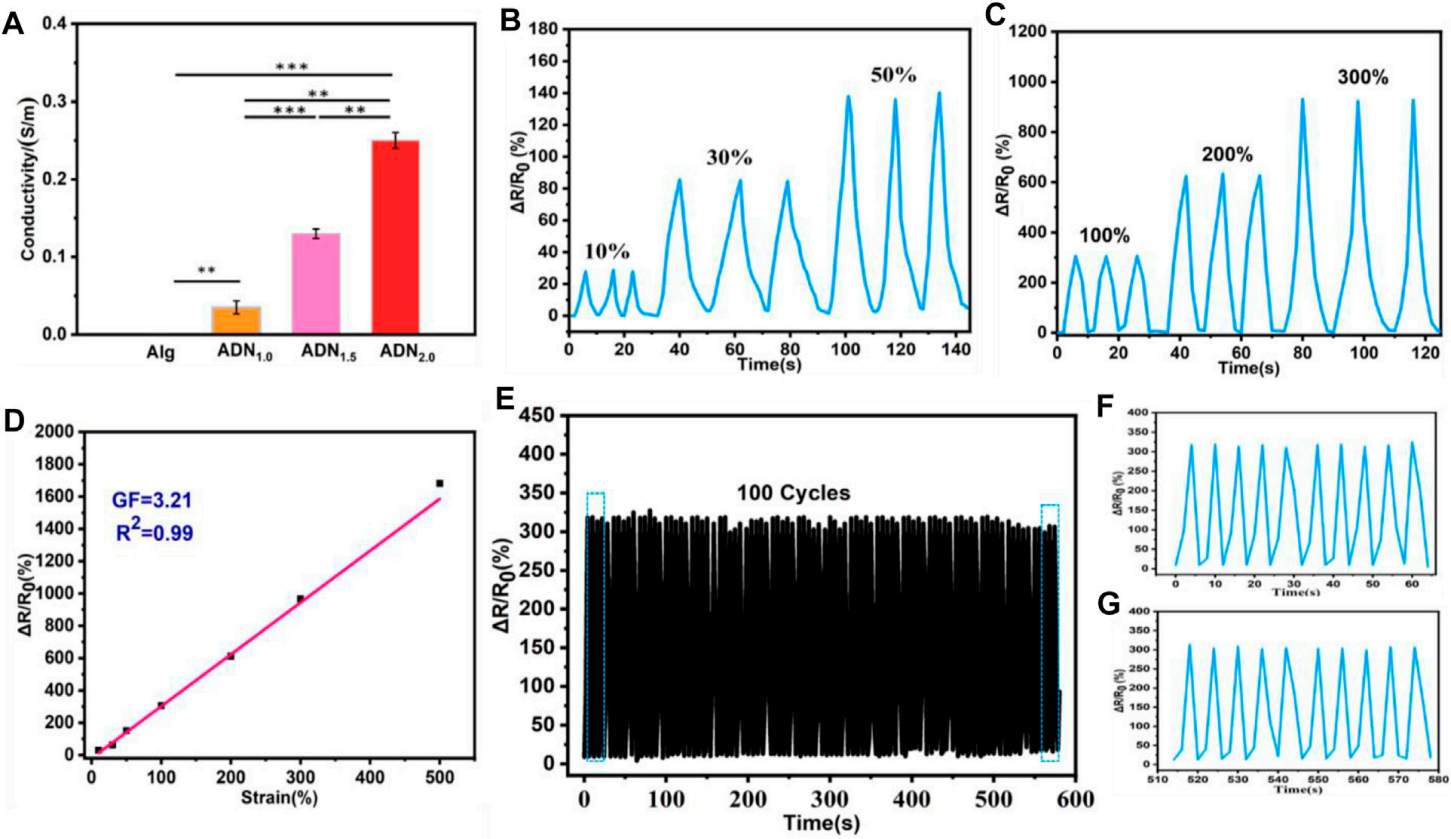
Electromechanical performances of the ADN_1.5_ hydrogel applicated as flexible strain sensor. **(A)** Conductivity comparison of hydrogels. The cyclic relative resistance changes of this hydrogel strain sensor under low strains **(B)** and high strains **(C)**, respectively. **(D)** The linear fitting of the relationship between the relative resistance changes and the strain of this hydrogel sensor. **(E)** The durability test of the hydrogel sensor under 0–100% strain. **(F)** Magnified signal during 1–10 cycles. **(G)** Magnified signal during 91–100 cycles.

The ADN hydrogel exhibited good electrical conductivity due to the introduction of the polyelectrolyte network. As shown in Figure 6A, compared with Alg hydrogel, the conductivity of ADN hydrogels was significantly improved. The conductivity of the composite hydrogel increased from 0.04 S/m to 0.25 S/m with the increase of NAS content from 1.0 wt% to 2.0 wt%. This might be due to the formation of more conductive pathways in the compound as the concentration of conductive ions increased. In order to study the variation in the resistance of the hydrogel with the tensile strain, the hydrogel was connected to the LCR meter to record the real-time resistance. The sensitivity of a strain sensor is expressed by the gauge factor (GF), which is calculated by the formula: GF = (ΔR/R_0_)/ε, where, ΔR = R−R_0_ (Chen et al., 2019; Xiong et al., 2020). Herein, R_0_ and R are the original resistance without strain and the real-time resistance, respectively, and ε is the applied strain. The change in the resistance rate (ΔR/R0) of three stretch-release cycles under low strain (10, 30, and 50%) and high strain (100, 200, and 300%) were shown in Figures 6B,C, respectively. It could be seen clearly that the resistance value of the hydrogel after three cyclic stretching (unstretched-stretched-unstretched) presented obvious and consistent cyclic change. As shown in Figure 6D, ΔR/R_0_ was linearly fitted to the strain and the GF of the ADN_1.5_ hydrogel was calculated to be 3.21. Compared with most reported flexible wearable strain sensors, the ADN hydrogel had higher sensitivity and stability in a wide range of strain changes. The ADN_1.5_ hydrogel-based strain sensor showed a stable response signal, when the strain was 100% (Figure 6E). The amplitude and waveform had tiny fluctuation after 100 consecutive loading and unloading cycles, proving the satisfactory stability and reliability of the strain sensor. The magnified signal during 1–10 cycles and 91–100 cycles were shown in Figures 6F,G, respectively.

### Application in Human Motions Monitoring

**FIGURE 7:**
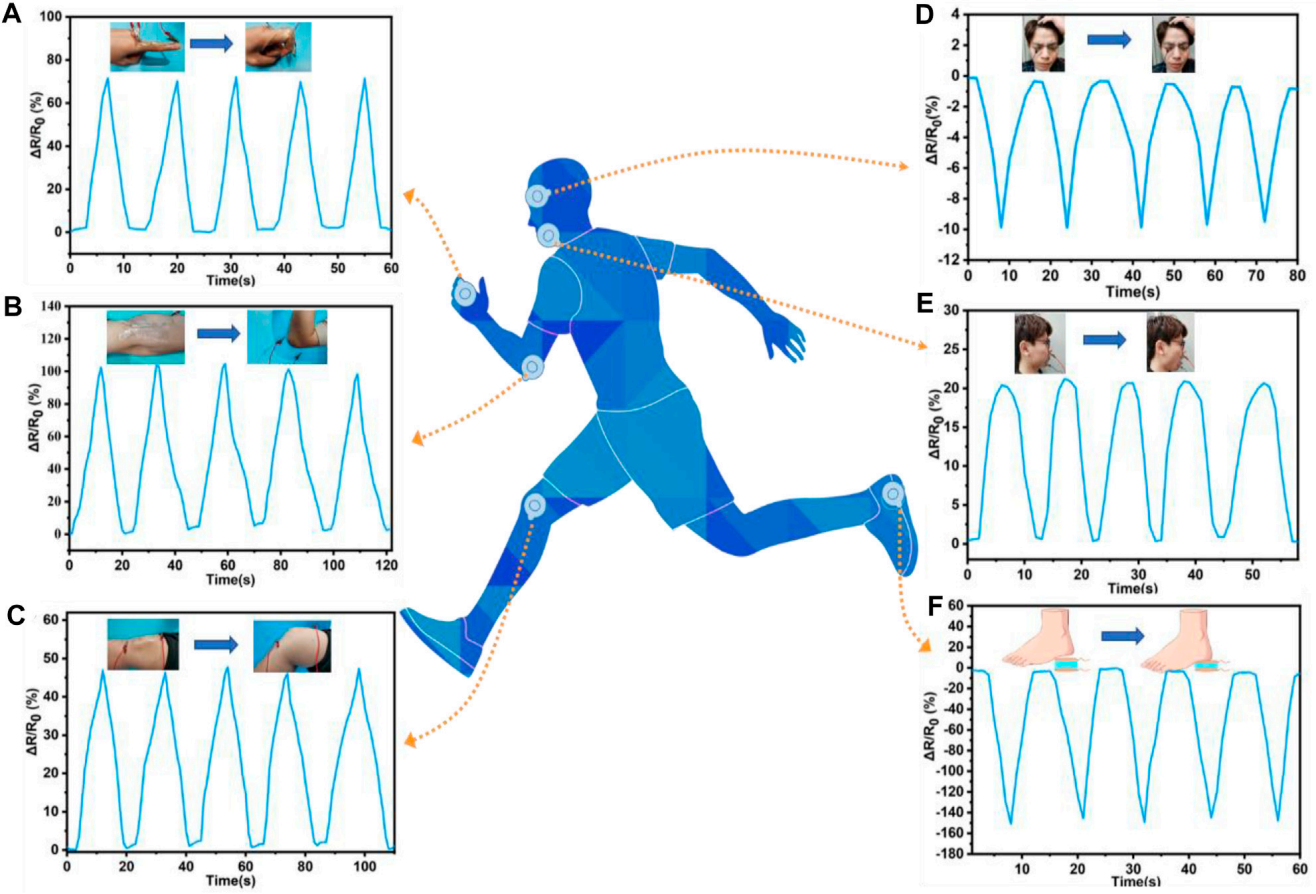
The real-time monitoring of human motions with ADN_1.5_ hydrogel-based strain sensors. The real-time monitoring of large-range human motions with finger **(A)**, elbow **(B)**, knee **(C)**. The real-time monitoring of tiny human motions: frown **(D)**, mouth movement **(E)**, and **(F)** foot planter pressure during locomotion.

The ADN hydrogel had the advantages of high sensitivity, fast response, wide sensing range, and good stability. Therefore, the wearable strain sensor based on the ADN hydrogel had a broad application prospect in human motion detection (Wang S. L. et al., 2022; Li et al., 2022). Herein, the ADN_1.5_ hydrogel was made into a wearable strain sensor, which was attached to the volunteer’s skin to monitor the movements of the human body from tiny deformations to large-scale motions in real-time. The ADN hydrogel-based strain sensor could accurately respond to full-range human motions, and it also had repeatable and stable signal output. In particular, the excellent optical transparency of the ADN_1.5_ hydrogel gave the strain sensor an unobtrusive visual appearance and allowed precise targeting of specific positions for real-time monitoring of human movements. The large-scale movement monitoring curves of human fingers, elbows, and knees were shown in Figure 7A–C. In the case of finger bending, when the finger bending angle gradually increased from 0° to 90°, the change of relative resistance increased from 0 to 72.5%. As the fingers returned from the bending to the stretching, the relative resistance also returned to the initial state. A stable repetitive response was observed during the five periodic scaling processes. In addition to detecting large body movements, the strain sensors could also detect subtle body movements. As shown in Figure 7D, our hydrogel-based strain sensor could detect tiny muscle movements around the eyebrow. When the volunteer performed periodic frowns exercises, a distinct and relatively uniform pattern of resistance was observed, displaying a wide prospect for facial recognition. And a unique and relatively consistent pattern of resistance was observed when the volunteer performed the periodic “opening-closing” movement (Figure 7E). In addition, a circular hydrogel disc was sandwiched between the sheet electrodes and placed directly under the heel to detect load during motion (Figure 7F). A steady repetitive response was observed during five periodic foot-up-and-down cycles. This application of the ADN hydrogel was feasible because of its excellent compressive strength, low mechanical hysteresis and electrical conductivity. With the unique design, the ADN hydrogel-based sensor could be used for plantar pressure measurements, and it also had great application potential in sports injury prevention, sports biomechanics, footwear design and research, and so on.

## Conclusion

In this study, we described a conductive double network hydrogel by introducing charge-rich polymorphic ions into the natural polysaccharide network. The ADN hydrogel exhibited remarkable stretchability, outstanding toughness, unique optical transmittance, self-healing, and general adhesion. In addition, we also designed a wearable strain sensor based on this ADN hydrogel. And the feasibility of monitoring human motions or analyzing human mental state based on signal acquisition of large joint flexion (such as finger, elbow, and knee) and local muscle movement (such as eyebrow and mouth) was demonstrated. Therefore, the highly stretchable, self-healing, and adhesive ADN hydrogel would be a promising material in aspects of human-machine interfaces, wearable monitoring systems and medical applications.

## Data Availability

The original contributions presented in the study are included in the article/Supplementary Material, further inquiries can be directed to the corresponding author/s.
