# Supplementary material for: Highly Transparent, Self-Healing, and Self-Adhesive Double Network Hydrogel for Wearable Sensors
Source: Front Bioeng Biotechnol. 2022 Feb 7;10:846401. doi: 10.3389/fbioe.2022.846401 (PMC8859421; doi:10.3389/fbioe.2022.846401)
Supplement: Supplementary file 1 [file DataSheet1.docx]

Supplementary materials

Highly transparent, self-healing, and self-adhesive double network

hydrogel for wearable sensors

*Kai Chen^1,4^, Mingxiang Liu^1^, Feng Wang^1^, Yunping Hu^1^, Pei Liu^1^, Cong Li^5^, Qianqian Du^5^, Yongsheng Yu^3*^, Xiufeng Xiao^1*^, Qian Feng^1,2*^*

*1 College of Chemistry and Materials Science, Fujian Provincial Key Laboratory of Advanced Materials Oriented Chemical Engineering, Fujian Normal University, Fuzhou, Fujian, China*

*2 Key Laboratory of Biorheological Science and Technology, Ministry of Education College of Bioengineering, Chongqing University, Chongqing, China*

*3 Chongqing Institute of Green and Intelligent Technology，Chinese Academy of Sciences*

*4 School of Resources and Chemical Engineering, Sanming University, Sanming, China*

*5 Department of Biomaterial, College of Life Sciences, Mudanjiang Medical University, Mudanjiang, China*

*Corresponding Author E-mail: Prof. Q. Feng (qianfeng@cqu.edu.cn);*

*Prof. X. F. Xiao (xfxiao@fjnu.edu.cn);*

*Prof. Y. S. Yu (yongshengy@126.com)*

**Table S1**. Formulations of the precursor solutions of the ADN hydrogels.

| sample | alginate  (wt %) | | NAS  (molL^-1^) | DAC  (mol/L) | EDTANa_2_Ca  (mmol/L) | GDL  (mmol/L) | V50  (mmol/L) |
| --- | --- | --- | --- | --- | --- | --- | --- |
| Alg | | 3 | 0 | 0 | 150 | 220 | 10 |
| ADN1.0 | | 3 | 1.0 | 1.5 | 150 | 220 | 10 |
| ADN1.5 | | 3 | 1.5 | 1.5 | 150 | 220 | 10 |
| ADN2.0 | | 3 | 2.0 | 1.5 | 150 | 220 | 10 |


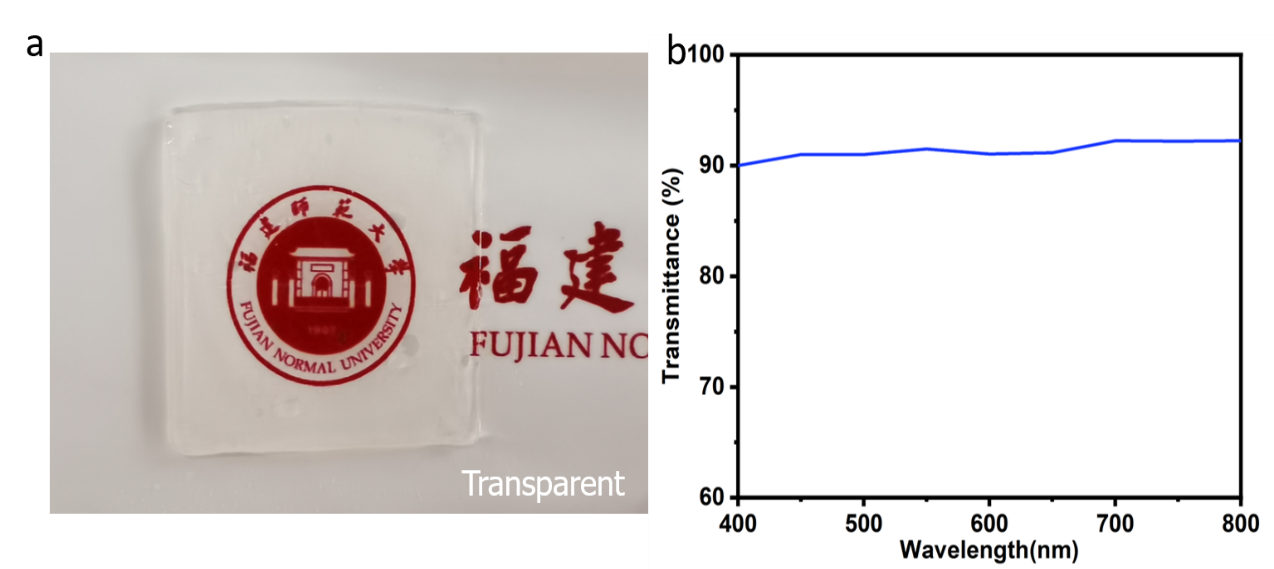


**Figure S1**. (a) Photograph representation of transparency. (b) Transmittance of the ADN_1.5_ hydrogel.


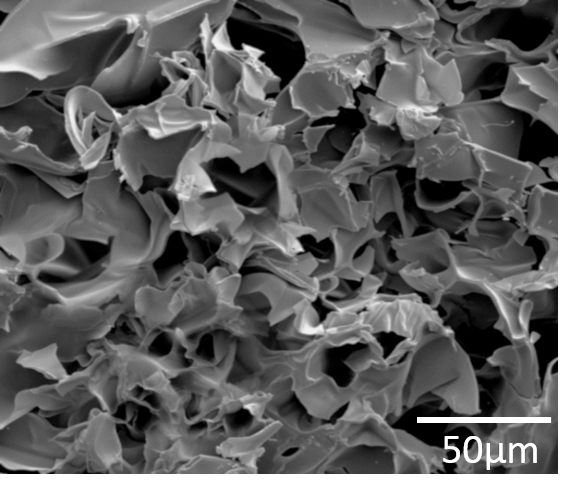


**Figure S2**. SEM image of the P(DAC-co-NAS) hydrogel.


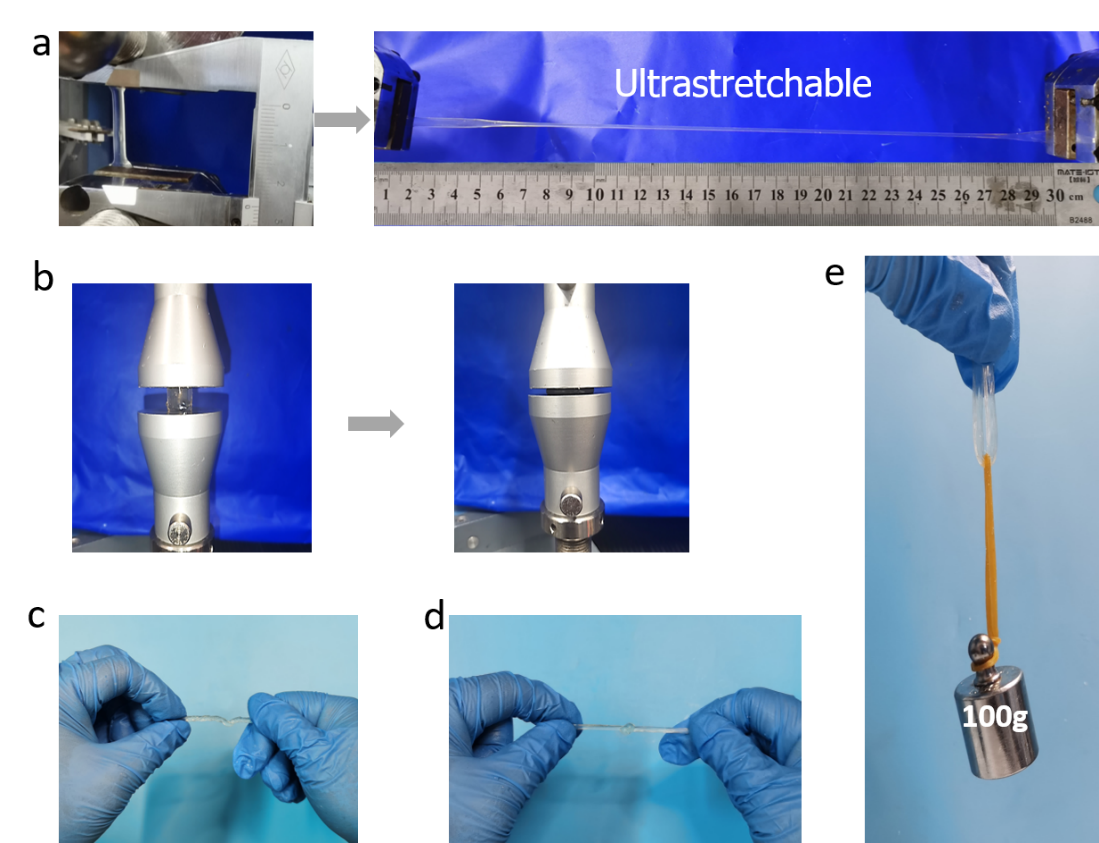


**Figure S3**. Photographs of the mechanical performances of the ADN_1.5_ hydrogels (a) stretching, (b) compression, (c) torsion. (d) knotting (e) a sample load weight of 100 g.
